# Optical Tracking and Digital Quantification of Beating Behavior in Bioengineered Human Cardiac Organoids

**DOI:** 10.3390/bios7030024

**Published:** 2017-06-23

**Authors:** Mahesh Devarasetty, Steven Forsythe, Thomas D. Shupe, Shay Soker, Colin E. Bishop, Anthony Atala, Aleksander Skardal

**Affiliations:** 1 Wake Forest Institute for Regenerative Medicine, Wake Forest School of Medicine, Medical Center Boulevard, Winston-Salem, NC 27157, USA; mdevaras@wakehealth.edu (M.D.); sforsyth@wakehealth.edu (S.F.); tshupe@wakehealth.edu (T.D.S.); ssoker@wakehealth.edu (S.S.); cbishop@wakehealth.edu (C.B.); aatala@wakehealth.edu (A.A.); 2 Virginia Tech-Wake Forest School of Biomedical Engineering and Sciences, Wake Forest School of Medicine, Winston-Salem, NC 27157, USA; 3 Department of Cancer Biology, Wake Forest School of Medicine, Medical Center Boulevard, Winston-Salem, NC 27157, USA; 4 Comprehensive Cancer Center at Wake Forest Baptist Medical, Medical Center Boulevard, Winston-Salem, NC 27157, USA

**Keywords:** organoid, organ-on-a-chip, heartbeat, biosensing, drug response, cardiomyocytes, cardiac organoids

## Abstract

Organoid and organ-on-a-chip technologies are rapidly advancing towards deployment for drug and toxicology screening applications. Liver and cardiac toxicities account for the majority of drug candidate failures in human trials. Liver toxicity generally produces liver cell death, while cardiac toxicity causes adverse changes in heart beat kinetics. In traditional 2D cultures, beating kinetics can be measured by electrode arrays, but in some 3D constructs, quantifying beating kinetics can be more challenging. For example, real time measurements of calcium flux or contractile forces are possible, yet rather complex. In this communication article, we demonstrate a simple sensing system based on software code that optically analyzes video capture files of beating cardiac organoids, translates these files in representations of moving pixels, and quantifies pixel movement activity over time to generate beat kinetic plots. We demonstrate this system using bioengineered cardiac organoids under baseline and drug conditions. This technology offers a non-invasive, low-cost, and incredibly simple method for tracking and quantifying beating behavior in cardiac organoids and organ-on-a-chip systems for drug and toxicology screening.

## 1. Introduction

Bioengineered organoid models and organ-on-a-chip, also known as microphysiological systems, have rapidly advanced in recent years for applications such as disease modeling and pharmacology/toxicology screening [1,2]. These model systems take on a wide variety of form factors, ranging from spheroid-based tissues, 3D cell cultures in engineered 3D matrices, and bioprinted constructs. Further development of bioengineered organ systems [3,4], combined with advanced microfluidic hardware, has resulted in organ-on-a-chip platforms that produce physiologically relevant responses to drugs and toxins. These platforms take a variety of forms, from single cell analysis devices to complex integrated organoid systems that can be employed for drug testing, toxicology [5], high throughput screens, and disease modeling [6,7]. These platforms bring together a variety of parameters that allow better mimicry of in vivo conditions, including 3D architecture, circulation, and integration of multiple tissues within one platform [1]. While a variety of tissues have been represented in these organoid and organ-on-a-chip systems, liver and cardiac platforms dominate the current research and development landscape. This is due to the fact that liver and cardiac toxicities account for the majority of drug candidate failures in human trials [8,9,10]. Unfortunately, a lack of models that accurately predict human drug toxicity drives up the cost of drug development [11].

One of the most important considerations when designing microphysiological models for drug screening is the strategy for reporting on the effects of a drug on the target tissue. For liver, this is relatively straightforward; as toxicity is indicated by cell injury or death. A wide variety of commercially available assays may be employed to measure cell death and decreases in cell function [12]. With respect to microphysiological cardiac models, measurement of compromised cell function is more difficult. Generally, cardiac toxicity does not result in cardiomyocyte death, but rather, manifests as changes in heartbeat rate or beating kinetics [13]. In traditional 2D cultures, beating kinetics can be easily measured by multi-electrode array (MEA) [13]. However, in 3D model systems, quantifying beat kinetics can be more challenging. Because these models are 3D, they do not always lend themselves to the planar MEA format. For example, to employ a MEA for measuring beating kinetics of spheroids, one would have to seed the spheroid on the MEA, and allow the spheroid to adhere and spread over the substrate, at which point it is no longer a spheroid. However, measurement techniques for recording cardiac organoid beating kinetics do exist. For example, real time measurement of calcium flux or contractile force are possible [8,14], but are rather complex, requiring fluorescent probes tied to ion channel function, complex imaging hardware, or complicated force transducing devices. These methodologies require either direct manipulation of the cells themselves, or biofabrication of the tissue constructs in or around a device that measures force, neither of which are completely non-invasive and limit the range of application.

In this communication article, we demonstrate a simple cardiac beat kinetic sensing system based on software code that analyzes imaging of beating cardiac organoids, translates these files into representations of moving pixels, and quantifies pixel movement activity over time to generate beat kinetics plots. We demonstrate this system using bioengineered cardiac organoids under baseline and drug challenge conditions. This technology offers a non-invasive, incredibly simple method for tracking and quantifying beating behavior in cardiac organoids and organ-on-a-chip systems for drug and toxicology screening.

## 2. Materials and Methods

### 2.1. Organoid Formation, Viability, and Encapsulation in Hydrogel

Induced pluripotent stem cell-derived cardiomyocytes (iPSC CMs) were commercially sourced from Axiogenesis (cat. # COR.4U Cardiomyocytes). Human primary cardiac fibroblasts were commercially sourced from ScienCell (cat. # 6330). Prior to organoid formation, iPSC CMs were culture on tissue culture plastic for 48 h in COR.4U medium until cells began beating spontaneously. At this point, iPSC CMs were harvested using trypsin-EDTA (Hyclone, Logan, UT, USA) and suspended in cardiomyocyte maintenance medium (CMM, Stem Cell Theranostics, Redwood City, CA, USA). Fibroblasts were added as 10% of the total cell number, and the volume was adjusted to reach a cell density of 10,000 cells/mL. We pipetted 100 μL of cell suspension into each well of a non-adherent, round-bottom, 96-well plates to produce spheroids (#7007, Corning, Corning, NY, USA) to result in approximately 1000 cells/spheroid. Well plates were incubated and observed daily until spheroid formation, and then immediately used in experiments. 

LIVE/DEAD staining was used to assess viability. Organoids were washed in PBS and then stained with LIVE/DEAD viability/cytotoxicity kit (Life Technologies): 2 μL/mL ethidium homodimer-1 and 0.5 μL/mL calcein AM (diluted in PBS) for 45 min at room temperature, protected from light. Organoids were transferred to a depression glass slide (Erie Scientific) and then imaged using TCS LSI macro confocal microscope with 5× macro objective (Leica). 

Cardiac organoids were immobilized in a 3D hydrogel to improve optical tracking using a simple fibrin-gelatin two-part system. The first part was prepared by dissolving 30 mg/mL fibrinogen and 35 mg/mL gelatin in PBS, while the second part was prepared by 20 U/mL thrombin in PBS. Crosslinking into a hydrogel was achieved by covering the desired volume of the fibrinogen-gelatin solution with the thrombin solution, thereby initiating enzymatic fibrinogen cleavage and subsequent crosslinking.

### 2.2. Software Code

A custom MATLAB^®^ code was developed to analyze and quantify cardiac spheroid beating from previously recorded video. First, the video was input into MATLAB^®^ and a segment was isolated from the full-length video: 10-s segments were default for this study. Video samples were separated into frames (getVideoFrames.m), then cropped to isolate the spheroid of interest and minimize noise (getAndCrop.m). These cropped frames were then converted to greyscale images. A reference frame of the spheroid was chosen from the resting phase of a beat when a spheroid remained stationary. Each subsequent frame of the sample was then compared to the reference frame to determine differences, pixel by pixel; if the difference surpassed a preset threshold, it was considered a moving pixel. This preset threshold was set to exclude pixel changes due to noise. This value was originally set by trial and error, based on the fact that the camera has reasonably low noise and the spheroid movement is significantly more distinct. A binary map of pixel movement was then generated where 1 denotes movement and 0 denotes static. The average value of the difference map for each frame was then plotted to produce the final beating plot (analyzeDisp.m). In the beating plots, the y-axis shows pixel %, or the fraction of total pixels that have been marked as moving at any given point in time (x-axis). In these beating plots, the positive slope of a peak corresponds to contraction (many pixels are changing) and the negative slope of a peak corresponds to relaxation (pixels are returning to their resting value). 

### 2.3. Beating Kinetics Evaluation and Drug Response Studies

Cardiac organoids in hydrogel were given fresh CMM alone, or with one of a panel of well-studied compounds for stimulating increased heart rates or inducing decreases in heart rates. Specifically, the organoids were subjected to CMM alone, 100 μM isoproterenol (Sigma-Aldrich, St. Louis, MO, USA) for 30 min, 500 nM epinephrine (Sigma-Aldrich) for 15 min, 1 μM quinidine (Sigma-Aldrich) for 10 min, 100 nM astemizole (Sigma-Aldrich) for 30 min, or 100 nM ricin A chain (Sigma-Aldrich) for 30 min. Following each of these incubation periods, video files were captured using a Leica inverted microscope under brightfield. The resulting video file was then processed into digitized representations of moving pixels, after which the sum of moving pixels was plotted versus time. 10-s segments of these videos were employed for the visual plots presented in the Results.

### 2.4. Statistical Methods

The data are presented as the means of number of replicates ± the standard deviation. All experiments were performed with *n* = 3 or higher. Values were compared using Student’s *t*-test (2-tailed) with two sample unequal variance, and *p* < 0.01 or less was considered statistically significant.

## 3. Results

### 3.1. Cardiac Organoid Formation and Initial Assessment

**Figure 1:**
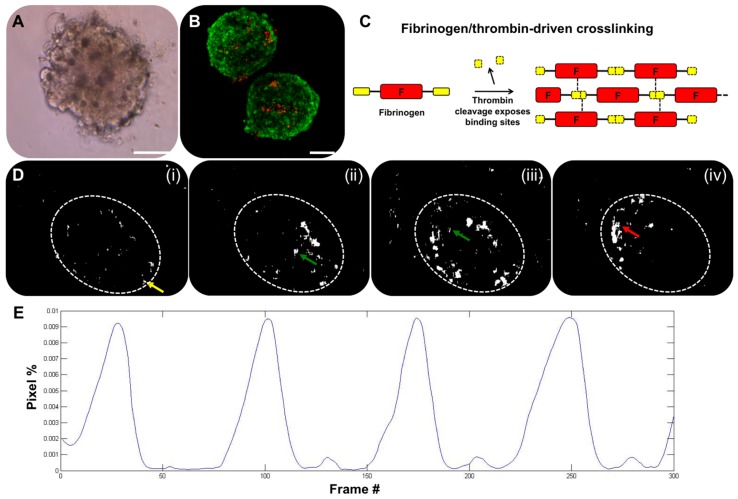
Overview of 3D cardiac organoids and heart rate analysis. (**A**) Organoids maintain a spherical, multi-cellular organization; and (**B**) can stay viable for over four weeks if necessary indicated by LIVE/DEAD viability/cytotoxicity staining. Green—viable calcein AM-stained cells; Red—ethidium homodimer-1-stained dead cells. Scale bar—100 μm; (**C**) Cartoon describing encapsulation in a fibrin hydrogel for immobilization during cardiac beat rate assessment. Fibrinogen is cleaved by thrombin, initiating formation of a fibrin hydrogel; (**D**) Frame grabs from a digitized video of a beating cardiac organoid. The overall organoid is indicated by the dotted white oval region. White indicates moving pixels. A single beat is identified in (i—iv): Yellow arrow—beat initiation; Green arrows—propagation of the beat across the organoid; Red arrow—culmination of the beat propagation at the opposite end of the organoid; (**E**) A plot showing quantification of the moving pixels as a percentage of total frame pixels over time, illustrating the heart rate kinetics.

We first developed cardiac organoids by inducing cell–cell aggregation in round bottom non-adherent 96-well plates. Briefly, these spherical cardiac organoids (Figure 1A) were formed using induced pluripotent stem (iPS) cell-derived cardiomyocytes that were cultured for four days prior to use in the subsequent studies. Following the initial aggregation period, the resulting organoids reached stable diameters of approximately 250 μm and began spontaneously beating, as indicated by cyclic contraction of the organoids. These organoids remained viable in culture for over four weeks (Figure 1B) and continually beat over that timeframe. As described above, standard methods for tracking and recording beat kinetics, such as MEA, are not always amenable to use in 3D systems. In the case of organoids with a spheroid architecture, the point of contact between the organoid and the MEA substrate would be minor, and measurements would not represent the contribution of the majority of the organoid. One solution is to allow the organoid to adhere and spread out over the MEA substrate, but by doing this, one has significantly changed the organoid form, including potential cell–cell interactions. Moreover, measurement of calcium flux by fluorescent dyes is transient and not suitable for long-term studies. As such, other methods for completely non-invasive measurement would be useful for studies in which the beating kinetics of 3D cardiac organoids is the primary output metric. To this end, we have developed a straightforward visual tracking system that provides quantitative data that is perhaps not as precise as MEA, but provides a quick data output in a non-invasive manner.

### 3.2. Optical Tracking and Organoid Heartbeat Quantification

**Figure 2:**
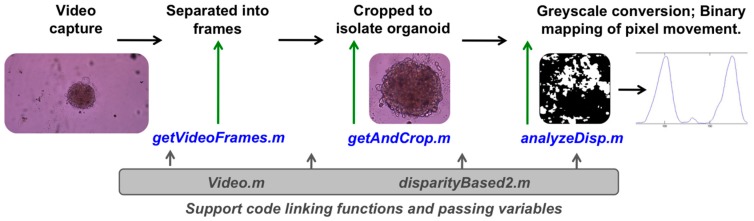
Flowchart describing the implementation of each code file during the heart rate quantification workflow. After executing the code in MATLAB^®^, video files are separated into frames using the function getVideoFrames.M. These frames are cropped to isolate the organoid using the function getAndCrop.m. Lastly, the function analyzeDisp.m is employed to convert frames to greyscale, perform binary mapping of pixel movement, and create a plot of quantity of moving pixels versus time, which illustrates the beating kinetics of the organoid. MATLAB^®^ functions Video.m and disparityBased2.m are employed to link the other functions and to pass variables from one function to another.

To capture and quantify the beating kinetics of cardiac organoids (or control cardiomyocyte monolayers), organoids were first suspended in a fibrin–gelatin hydrogel to immobilize the organoids during subsequent tests (Figure 1C). This substrate did not affect organoid beating. During culture of cardiac constructs, video of beating organoids was captured by standard brightfield microscopy. Video files were analyzed using custom written MATLAB^®^ code (Data File 1), which utilizes a series of MATLAB^®^ functions (Data Files 2–6). The software created a reference frame, based on a frame of the video during the resting frame of the beat, and compared pixels in each subsequent frame, determining which pixels represented movement over time. The moving pixels in each frame were then used to generate a black and white binarized video representation of the organoid beating behavior, allowing (1) visualization of beat propagation (Figure 1D); and (2) quantification of the total number of moving pixels versus time and generation of a plot showing these kinetics along with beat rate (Figure 1E). A flow chart of how these pieces of code are implemented in this workflow is described in Figure 2.

### 3.3. Modulation of Organoid Beat Rate through Drug and Toxin Exposure

To further assess the utility of this cardiac beating tracking tool, a panel of drugs and compounds with documented effects on cardiomyocyte beating function were screened. These drugs were used to alter the heart rate of the organoids in order demonstrate the utility of employing the software described above for automated heart rate quantification. Cardiac organoids were incubated under baseline conditions (no drug); with epinephrine (a hormone that binds to a variety of adrenergic receptors, including the β-adrenergic receptors of cardiomyocytes and stimulates increase heart rate); with isoproterenol (a non-selective β-adrenergic receptor agonist often used clinically for treatment of bradycardia); with quinidine (a class 1 antiarrhythmic agent that blocks fast inward sodium current); with astemizole (an antihistamine withdrawn from the market due to causing QT interval prolongation and arrhythmias in the heart); and with ricin A chain (one of the two chains comprising the severely toxic agent ricin). Following incubation video files were captured for analysis.

**Figure 3:**
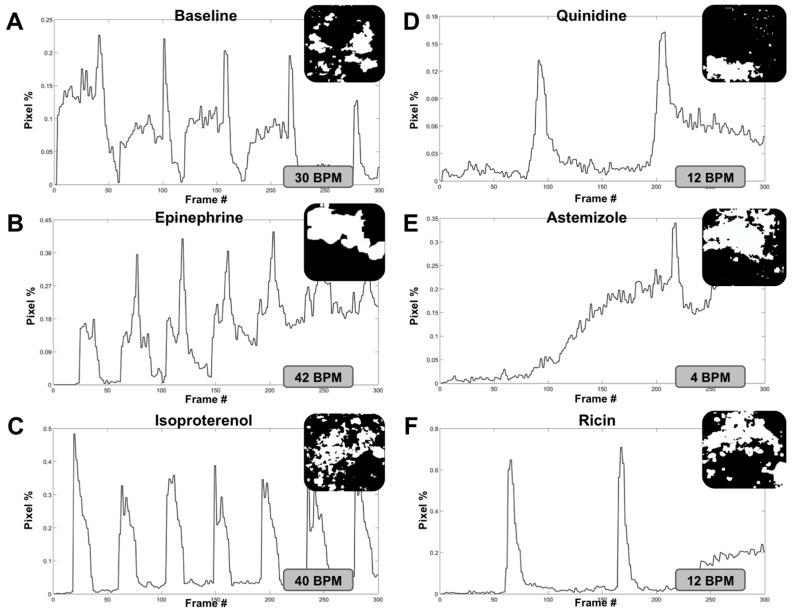
Analysis of cardiac organoid heart rate corresponds with changes induced using a panel of compounds that stimulate or attenuate heart rate. Cardiac organoid beat plots consisting of pixel movement in relation to the initial digitized video frame versus time, frame grabs of each digitized pixel movement video, and subsequent calculated heart rate under (**A**) baseline conditions; and after incubation with (**B**) epinephrine; (**C**) isoproterenol; (**D**) quinidine; (**E**) astemizole; and (**F**) ricin A chain. Each beat plot shows a 10-s segment of the longer video files that were captured.

**Figure 4:**
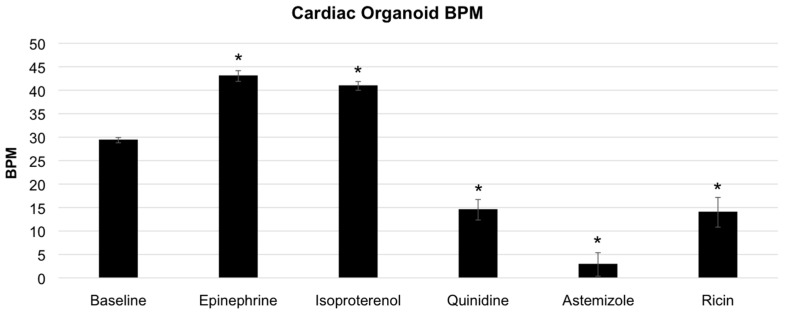
Summary graph of cardiac organoid heart rates under baseline and drug compound conditions. Cardiac organoids showed statistically significant increases from baseline conditions under incubation with epinephrine or isoproterenol. Additionally, cardiac organoids showed statistically significant decreases from baseline conditions under incubation with quinidine, astemizole, or ricin. Statistical significance: * *p* < 0.01 compared to baseline.

Following video capture under each condition, individual video files were analyzed using the MATLAB^®^ code and functions described above, resulting in digitized video files and subsequent pixel activity plots. Frame captures from the digitized video files and 10 s segments (300 frames) of each pixel activity plot (or beating activity plot) are shown in Figure 3. For these experiments, baseline beat rate was 30 beats per minute (BPM) (Figure 3A). Both isoproterenol and epinephrine induced increases in organoid beat rates, as expected. Specifically, epinephrine increased the beat rate to 42 BPM (Figure 3B), while isoproterenol incubation increased the beat rate to 40 BPM (Figure 3C). Conversely, quinidine, astemizole, and ricin A chain all induced decreases in organoid beat rates following incubation with each agent, also as expected. Specifically, quinidine decreased the beat rate to 12 BPM (Figure 3D), astemizole decreased the beat rate to 4 BPM (Figure 3E), and ricin A chain decreased the beat rate to 12 BPM (Figure 3F). These outcomes are summarized in Figure 4. Importantly, we observed statistical significance between baseline conditions compared to each drug that increased BPM and each drug that decreased BPM (*p* < 0.01).

### 3.4. Accuracy Comparison—Digital Quantification Versus Manual Counting

Next, we determined the beat rates for each of the videos under the different incubation conditions by simply counting beats manually. These results were then tabulated in order to compare with results generated by the software code-generated data. These data are presented in Table 1, including drug concentration and exposure time, video length, number of manually observed beats, observed effective heart rate, digitized heart rate, and a calculated accuracy percentage. Under baseline, isoproterenol, astemizole, and ricin A chain incubations, we found a 100% correlation between the digital quantification and our manual observations. Under epinephrine incubation, there was a 99.6% correlation between these two methodologies. This minor discrepancy is simply due to the fact that the video length was 33 s rather than 30 s, resulting in a slight offset after calculating the BPM rate for 60 s. Under quinidine incubation, we found a more significant discrepancy. The digitized quantification gave 12 BPM while our manually observed result was 16 BPM, resulting in a 75% correlation. This result likely stems from the fact that the visible magnitude of each beat under quinidine treatment is very low, potentially resulting in some beats that do not register amid noise in the system. Despite this discrepancy, we still have an impressive overall correlation of 95.76%, suggesting that this is a relatively consistent method for automating cardiac organoid (or cardiomyocyte culture) beat tracking using a non-invasive optical sensing system. 

## 4. Discussion

Three-dimensional tissue organoid systems and organ-on-a-chip systems have demonstrated immense potential in the drug development and toxicology screening field [1,15,16,17]. Success in bringing new drugs to market hinges on being able to consistently detect instances of toxicity in human cell-based models using technologies that are (1) accurate; and (2) not prohibitively expensive. Our team, and others, have focused initial work on bioengineering model systems using human cells that excel in recapitulating certain aspects of human physiology, in as simple a manner as possible, while retaining key functions such as accurate responses to drugs and toxins. Simple systems with these capabilities, paired with methods for biosensing and detection of such drug responses [18,19], have the potential to be seamlessly integrated into the drug development pipeline; thus improving the chances of new and effective drug candidates to successfully navigate regulatory hurdles. Equally as important, if these systems are sensitive to toxic effects, the potential also exists to identify toxic drug candidates earlier in the drug development pipeline, indicating failure during the early stages rather than during clinical trials. This would substantially reduce overall costs involved in bringing a new drug to market [11,20]. 

We believe that the simple, inexpensive, and cross-platform compatible software approach described in this communication has the potential to be employed for this purpose. We were able to accurately quantify 3D cardiac organoid beat rates in response to six different conditions, including heart rate stimulating agents and heart rate decreasing toxic agents. Importantly, the described method can be adapted for any video capture system. We used a simple brightfield microscope-mounted camera to capture video of the cardiac organoids in each condition. However, this technique is adaptable to nearly any form factor of culture chamber and video capture, allowing for widespread use. Early testing of this software was conducted on 2D rat cardiomyocyte cultures—a long held industry standard cardiac model—with great success (not shown). However, we shifted to human iPS derived 3D organoids to be more representative of human biology. Additionally, we have recently integrated our analysis platform with a heart-on-a-chip system complete with circulating perfusion and an onboard camera biosensor that may be linked in series with other organoid types for integrated multi-organ representative drug response analysis. Moreover, as these in vitro model systems continue to advance, we envision this and other biosensing technologies as being implemented not only in drug development and toxicology screening, but also in other applications requiring real-time analysis of physiology, including disease modeling by induction of disease states within these models, and personalized medicine applications such as identifying the most effective, and also the safest, therapy for a particular patient through use of their own cells. In all of these applications, accurate, inexpensive, and cross-platform compatible approaches will rapidly advance these technologies and their deployment in commercial and clinical settings.

## Figures and Tables

**Table 1:** Summary of the drug conditions (concentration and exposure time), video details, manually observed organoid beats, observed effective heart rate, digitally quantified heart rate, and accuracy between the manual and automated analyses.

Condition	Drug Concentration	Exposure Time (min)	Average Video Time (s)	Average Observed Beats	Average Observed Effective Heart Rate (BPM)	Average Digitized Heart Rate (BPM)	Accuracy
Baseline	n/a	n/a	30	15	30	30	100%
Isoproterenol	100 uM	30	21	14	40	40	100%
Epinephrine	500 nM	15	33	23	41.8	42	99.6%
Quinidine	1 uM	10	15	4	16	12	75%
Astemizole	100 nM	30	30	2	4	4	100%
Ricin A Chain	100 nM	30	30	6	12	12	100%

## Supplementary Materials

The following are available online at www.mdpi.com/2079-6374/7/3/24/s1, Data File 1. MATLAB code for pixelation and beat plot output—Executable code.txt, Data File 2. MATLAB function file—analyzeDisp.m, Data File 3. MATLAB function file—disparityBased2.m, Data File 4. MATLAB function file—getAndCrop.m, Data File 5. MATLAB function file—getVideoFrames.m, Data File 6. MATLAB function file—Video.m.
